# *cis*-Bis(4-methyl­piperazine-1-carbo­dithio­ato-κ^2^ *S*,*S*′)bis­(pyridine-κ*N*)cadmium

**DOI:** 10.1107/S1600536811054791

**Published:** 2011-12-23

**Authors:** P. Valarmathi, S. Thirumaran, Kamini Kapoor, Vivek K. Gupta, Rajni Kant

**Affiliations:** a Department of Chemistry, Annamalai University, Annamalainagar 608 002, India; b X-ray Crystallography Laboratory, Post-Graduate Department of Physics & Electronics, University of Jammu, Jammu Tawi 180 006, India

## Abstract

In the title complex, [Cd(C_6_H_11_N_2_S_2_)_2_(C_5_H_5_N)_2_], the Cd^II^ ion is hexa­coordinated by two N atoms from two pyridine ligands and by four S atoms from two dithio­carbamate ligands in a distorted octa­hedral geometry. The Cd^II^ ion lies on a twofold axis. The piperazine ring is in chair conformation and its least-squares plane makes a dihedral angle of 81.4 (1)° with that of the pyridine ring.

## Related literature

For background to and applications of dithio­carbamates, see: Bessergenev *et al.* (1997 ▶); Havel (1975 ▶); Valarmathi *et al.* (2011 ▶); Pickett & O’Brien (2001 ▶). For related structures, see: Ivanov *et al.* (2006 ▶); Onwudiwe & Ajibade (2010 ▶); Yin *et al.* (2004 ▶).
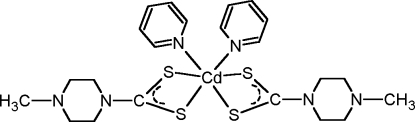


## Experimental

### 

#### Crystal data

[Cd(C_6_H_11_N_2_S_2_)_2_(C_5_H_5_N)_2_]
*M* *_r_* = 621.18
Monoclinic, 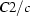

*a* = 17.7065 (7) Å
*b* = 8.7806 (6) Å
*c* = 20.6171 (8) Å
β = 122.276 (5)°
*V* = 2710.1 (2) Å^3^
*Z* = 4
Mo *K*α radiation
μ = 1.14 mm^−1^
*T* = 293 K
0.3 × 0.2 × 0.2 mm

#### Data collection

Oxford Diffraction Xcalibur Sapphire3 diffractometer
Absorption correction: multi-scan (*CrysAlis RED*; Oxford Diffraction, 2010 ▶) *T*_min_ = 0.645, *T*_max_ = 1.000
24135 measured reflections
2383 independent reflections
2088 reflections with *I* > 2σ(*I*)
*R*_int_ = 0.047

#### Refinement

*R*[*F* ^2^ > 2σ(*F* ^2^)] = 0.026
*wR*(*F* ^2^) = 0.057
*S* = 1.07
2383 reflections
151 parameters
H-atom parameters constrained
Δρ_max_ = 0.40 e Å^−3^
Δρ_min_ = −0.30 e Å^−3^

### 

Data collection: *CrysAlis PRO* (Oxford Diffraction, 2010 ▶); cell refinement: *CrysAlis PRO*; data reduction: *CrysAlis RED* (Oxford Diffraction, 2010 ▶); program(s) used to solve structure: *SHELXS97* (Sheldrick, 2008 ▶); program(s) used to refine structure: *SHELXL97* (Sheldrick, 2008 ▶); molecular graphics: *ORTEP-3* (Farrugia, 1997 ▶); software used to prepare material for publication: *PLATON* (Spek, 2009 ▶) and *PARST* (Nardelli, 1995 ▶).

## Supplementary Material

Crystal structure: contains datablock(s) I, global. DOI: 10.1107/S1600536811054791/nc2258sup1.cif

Structure factors: contains datablock(s) I. DOI: 10.1107/S1600536811054791/nc2258Isup2.hkl

Additional supplementary materials:  crystallographic information; 3D view; checkCIF report

## Figures and Tables

**Fig. 1.**
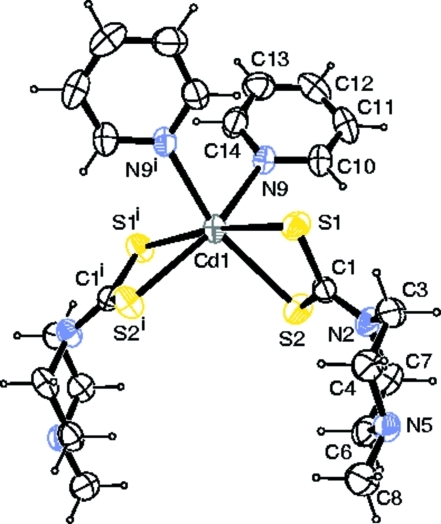
ORTEP view of the molecule with the atom-labeling scheme. The thermal ellipsoids are drawn at the 40% probability level. H atoms are shown as small spheres of arbitrary radii. Symmetry code = -x, y, -z + 1/2.

## supplementary crystallographic information

### Comment

The use of nitrogen donor adducts of cadmium dithiocarbamate complexes as convenient synthetic precursors for cadmium sulfide nanoparticles (Bessergenev *et al.*, 1997; Havel, 1975, Pickett & O'Brien 2001; Valarmathi *et al.*, 2011), attract continued attention to adducts of cadmium dithiocarbamates. As part of an on-going structural studies of nitrogen donor adducts of cadmium dithiocarbamates, the analysis of the title compound, (I), was undertaken. The bond angles around the cadmium atom are in the range of 67.56 (2) to 171.50 (3)°. The Cd—S bond lengths are: CD1—S1 = 2.6621 (7); CD1—S2 = to 2.6803 (7) Å and are in good agreement with those reported for other Cd- dithiocarbonato complexes (Ivanov *et al.*, 2006; Onwudiwe *et al.*, 2010; Yin *et al.*, 2004). The piperazine ring has a chair conformation. The asymmetry parameters are: ΔCs(N2)=0.72; ΔC2(N2—C3)= 0.73. The dihedral angle between the best least squares planes through piperazine and pyridine rings is 81.4 (1)°.

### Experimental

Cd(4-mpzdtc)_2_] (1 mmol, 0.483 g) was dissolved in 50 ml of warm pyridine. The yellow solution obtained was filtered and kept for evaporation. After few days, single crystals suitable for X-ray structural analysis were obtained (m.p. 552–554 K).

### Refinement

All H atoms were positioned geometrically and were treated as riding on their parent C atoms, with C—H distances of 0.93–0.97 Å and with *U*_iso_(H) = 1.2U_eq_(C) or 1.5U_eq_ for methyl H atoms.

### Crystal data

**Table:**

[Cd(C_6_H_11_N_2_S_2_)_2_(C_5_H_5_N)_2_]	*F*(000) = 1272
*M**_r_* = 621.18	*D*_x_ = 1.522 Mg m^−^^3^
Monoclinic, *C*2/*c*	Mo *K*α radiation, λ = 0.71073 Å
Hall symbol: -C 2yc	Cell parameters from 11642 reflections
*a* = 17.7065 (7) Å	θ = 3.5–29.1°
*b* = 8.7806 (6) Å	µ = 1.14 mm^−^^1^
*c* = 20.6171 (8) Å	*T* = 293 K
β = 122.276 (5)°	Block, white
*V* = 2710.1 (2) Å^3^	0.3 × 0.2 × 0.2 mm
*Z* = 4	

### Data collection

**Table:**

Oxford Diffraction Xcalibur Sapphire3 diffractometer	2383 independent reflections
Radiation source: fine-focus sealed tube	2088 reflections with *I* > 2σ(*I*)
graphite	*R*_int_ = 0.047
Detector resolution: 16.1049 pixels mm^-1^	θ_max_ = 25.0°, θ_min_ = 3.8°
ω scans	*h* = −20→20
Absorption correction: multi-scan (*CrysAlis RED*; Oxford Diffraction, 2010)	*k* = −10→10
*T*_min_ = 0.645, *T*_max_ = 1.000	*l* = −24→24
24135 measured reflections	

### Refinement

**Table:**

Refinement on *F*^2^	Primary atom site location: structure-invariant direct methods
Least-squares matrix: full	Secondary atom site location: difference Fourier map
*R*[*F*^2^ > 2σ(*F*^2^)] = 0.026	Hydrogen site location: inferred from neighbouring sites
*wR*(*F*^2^) = 0.057	H-atom parameters constrained
*S* = 1.07	*w* = 1/[σ^2^(*F*_o_^2^) + (0.0157*P*)^2^ + 4.0129*P*] where *P* = (*F*_o_^2^ + 2*F*_c_^2^)/3
2383 reflections	(Δ/σ)_max_ < 0.001
151 parameters	Δρ_max_ = 0.40 e Å^−^^3^
0 restraints	Δρ_min_ = −0.30 e Å^−^^3^

### Special details

**Table:**

Experimental. *CrysAlis PRO*, Oxford Diffraction Ltd., Version 1.171.34.40 (release 27–08-2010 CrysAlis171. NET) (compiled Aug 27 2010,11:50:40) Empirical absorption correction using spherical harmonics, implemented in SCALE3 ABSPACK scaling algorithm.
Geometry. All e.s.d.'s (except the e.s.d. in the dihedral angle between two l.s. planes) are estimated using the full covariance matrix. The cell e.s.d.'s are taken into account individually in the estimation of e.s.d.'s in distances, angles and torsion angles; correlations between e.s.d.'s in cell parameters are only used when they are defined by crystal symmetry. An approximate (isotropic) treatment of cell e.s.d.'s is used for estimating e.s.d.'s involving l.s. planes.
Refinement. Refinement of *F*^2^ against ALL reflections. The weighted *R*-factor *wR* and goodness of fit *S* are based on *F*^2^, conventional *R*-factors *R* are based on *F*, with *F* set to zero for negative *F*^2^. The threshold expression of *F*^2^ > σ(*F*^2^) is used only for calculating *R*-factors(gt) *etc*. and is not relevant to the choice of reflections for refinement. *R*-factors based on *F*^2^ are statistically about twice as large as those based on *F*, and *R*- factors based on ALL data will be even larger.

### Fractional atomic coordinates and isotropic or equivalent isotropic displacement parameters (Å^2^)

**Table:**

	*x*	*y*	*z*	*U*_iso_*/*U*_eq_	
Cd1	0.0000	0.14900 (3)	0.2500	0.04703 (10)	
S1	0.08961 (5)	0.12653 (8)	0.17998 (4)	0.05259 (19)	
S2	−0.07368 (5)	−0.05272 (9)	0.13462 (4)	0.0550 (2)	
C1	0.01848 (16)	−0.0220 (3)	0.12909 (13)	0.0415 (6)	
N2	0.03409 (14)	−0.1100 (3)	0.08483 (12)	0.0472 (5)	
C3	−0.01499 (18)	−0.2496 (3)	0.04814 (16)	0.0552 (7)	
H3A	−0.0316	−0.2518	−0.0049	0.066*	
H3B	−0.0692	−0.2532	0.0490	0.066*	
C4	0.04322 (18)	−0.3850 (3)	0.09059 (16)	0.0537 (7)	
H4A	0.0569	−0.3849	0.1428	0.064*	
H4B	0.0109	−0.4780	0.0660	0.064*	
N5	0.12635 (14)	−0.3821 (3)	0.09173 (12)	0.0486 (5)	
C6	0.17350 (18)	−0.2393 (3)	0.12557 (16)	0.0535 (7)	
H6A	0.2272	−0.2361	0.1239	0.064*	
H6B	0.1913	−0.2354	0.1789	0.064*	
C7	0.11636 (19)	−0.1028 (3)	0.08401 (17)	0.0552 (7)	
H7A	0.1487	−0.0101	0.1089	0.066*	
H7B	0.1018	−0.1019	0.0315	0.066*	
C8	0.1830 (2)	−0.5113 (4)	0.13378 (18)	0.0658 (8)	
H8A	0.2363	−0.5078	0.1323	0.099*	
H8B	0.1514	−0.6042	0.1107	0.099*	
H8C	0.1988	−0.5070	0.1861	0.099*	
N9	−0.09469 (14)	0.3546 (3)	0.16984 (11)	0.0476 (5)	
C10	−0.13114 (18)	0.3577 (4)	0.09431 (15)	0.0561 (7)	
H10	−0.1166	0.2808	0.0718	0.067*	
C11	−0.1891 (2)	0.4693 (4)	0.04841 (17)	0.0701 (9)	
H11	−0.2141	0.4665	−0.0043	0.084*	
C12	−0.2098 (2)	0.5841 (4)	0.0804 (2)	0.0707 (9)	
H12	−0.2492	0.6608	0.0501	0.085*	
C13	−0.1715 (2)	0.5845 (4)	0.1582 (2)	0.0702 (9)	
H13	−0.1833	0.6625	0.1821	0.084*	
C14	−0.1153 (2)	0.4675 (4)	0.20016 (17)	0.0625 (8)	
H14	−0.0902	0.4674	0.2529	0.075*	

### Atomic displacement parameters (Å^2^)

**Table:**

	*U*^11^	*U*^22^	*U*^33^	*U*^12^	*U*^13^	*U*^23^
Cd1	0.05092 (17)	0.05401 (19)	0.03837 (15)	0.000	0.02532 (13)	0.000
S1	0.0537 (4)	0.0546 (4)	0.0574 (4)	−0.0160 (3)	0.0350 (4)	−0.0129 (3)
S2	0.0477 (4)	0.0652 (5)	0.0606 (4)	−0.0153 (3)	0.0346 (4)	−0.0175 (4)
C1	0.0434 (14)	0.0443 (15)	0.0351 (13)	0.0004 (12)	0.0198 (11)	0.0044 (11)
N2	0.0480 (12)	0.0535 (14)	0.0487 (12)	−0.0072 (10)	0.0317 (11)	−0.0095 (10)
C3	0.0494 (16)	0.066 (2)	0.0482 (16)	−0.0072 (14)	0.0247 (14)	−0.0184 (14)
C4	0.0593 (17)	0.0576 (18)	0.0497 (16)	−0.0190 (14)	0.0330 (14)	−0.0141 (13)
N5	0.0534 (13)	0.0503 (14)	0.0459 (12)	−0.0031 (11)	0.0291 (11)	−0.0021 (10)
C6	0.0502 (16)	0.0602 (18)	0.0599 (17)	−0.0077 (14)	0.0360 (14)	−0.0033 (15)
C7	0.0668 (18)	0.0536 (17)	0.0679 (19)	−0.0057 (14)	0.0511 (16)	−0.0017 (14)
C8	0.0697 (19)	0.060 (2)	0.0642 (19)	0.0003 (16)	0.0330 (16)	0.0036 (16)
N9	0.0469 (12)	0.0577 (14)	0.0362 (11)	0.0016 (11)	0.0208 (10)	−0.0025 (11)
C10	0.0576 (17)	0.072 (2)	0.0430 (15)	−0.0001 (16)	0.0295 (14)	−0.0007 (15)
C11	0.065 (2)	0.096 (3)	0.0455 (17)	0.0059 (19)	0.0269 (16)	0.0226 (18)
C12	0.0588 (19)	0.072 (2)	0.081 (2)	0.0091 (17)	0.0365 (18)	0.034 (2)
C13	0.078 (2)	0.0545 (19)	0.080 (2)	0.0082 (17)	0.0436 (19)	0.0018 (17)
C14	0.071 (2)	0.063 (2)	0.0449 (16)	0.0074 (16)	0.0251 (15)	−0.0054 (15)

### Geometric parameters (Å, °)

**Table:**

Cd1—N9^i^	2.417 (2)	C6—C7	1.506 (4)
Cd1—N9	2.417 (2)	C6—H6A	0.9700
Cd1—S1^i^	2.6621 (7)	C6—H6B	0.9700
Cd1—S1	2.6621 (7)	C7—H7A	0.9700
Cd1—S2	2.6803 (7)	C7—H7B	0.9700
Cd1—S2^i^	2.6803 (7)	C8—H8A	0.9600
S1—C1	1.725 (3)	C8—H8B	0.9600
S2—C1	1.717 (2)	C8—H8C	0.9600
C1—N2	1.333 (3)	N9—C14	1.323 (3)
N2—C3	1.459 (3)	N9—C10	1.330 (3)
N2—C7	1.467 (3)	C10—C11	1.368 (4)
C3—C4	1.510 (4)	C10—H10	0.9300
C3—H3A	0.9700	C11—C12	1.359 (5)
C3—H3B	0.9700	C11—H11	0.9300
C4—N5	1.460 (3)	C12—C13	1.368 (4)
C4—H4A	0.9700	C12—H12	0.9300
C4—H4B	0.9700	C13—C14	1.369 (4)
N5—C8	1.455 (4)	C13—H13	0.9300
N5—C6	1.460 (3)	C14—H14	0.9300
			
N9^i^—Cd1—N9	83.36 (10)	C4—N5—C6	109.7 (2)
N9^i^—Cd1—S1^i^	94.66 (5)	N5—C6—C7	111.9 (2)
N9—Cd1—S1^i^	91.69 (5)	N5—C6—H6A	109.2
N9^i^—Cd1—S1	91.69 (5)	C7—C6—H6A	109.2
N9—Cd1—S1	94.66 (5)	N5—C6—H6B	109.2
S1^i^—Cd1—S1	171.50 (3)	C7—C6—H6B	109.2
N9^i^—Cd1—S2	158.69 (5)	H6A—C6—H6B	107.9
N9—Cd1—S2	93.18 (5)	N2—C7—C6	109.2 (2)
S1^i^—Cd1—S2	106.48 (2)	N2—C7—H7A	109.8
S1—Cd1—S2	67.56 (2)	C6—C7—H7A	109.8
N9^i^—Cd1—S2^i^	93.18 (5)	N2—C7—H7B	109.8
N9—Cd1—S2^i^	158.69 (5)	C6—C7—H7B	109.8
S1^i^—Cd1—S2^i^	67.56 (2)	H7A—C7—H7B	108.3
S1—Cd1—S2^i^	106.48 (2)	N5—C8—H8A	109.5
S2—Cd1—S2^i^	97.27 (4)	N5—C8—H8B	109.5
C1—S1—Cd1	86.15 (8)	H8A—C8—H8B	109.5
C1—S2—Cd1	85.73 (9)	N5—C8—H8C	109.5
N2—C1—S2	120.42 (19)	H8A—C8—H8C	109.5
N2—C1—S1	120.25 (18)	H8B—C8—H8C	109.5
S2—C1—S1	119.31 (15)	C14—N9—C10	117.0 (2)
C1—N2—C3	123.9 (2)	C14—N9—Cd1	120.15 (18)
C1—N2—C7	123.6 (2)	C10—N9—Cd1	122.80 (19)
C3—N2—C7	110.5 (2)	N9—C10—C11	122.7 (3)
N2—C3—C4	109.1 (2)	N9—C10—H10	118.6
N2—C3—H3A	109.9	C11—C10—H10	118.6
C4—C3—H3A	109.9	C12—C11—C10	119.5 (3)
N2—C3—H3B	109.9	C12—C11—H11	120.2
C4—C3—H3B	109.9	C10—C11—H11	120.2
H3A—C3—H3B	108.3	C11—C12—C13	118.5 (3)
N5—C4—C3	111.5 (2)	C11—C12—H12	120.7
N5—C4—H4A	109.3	C13—C12—H12	120.7
C3—C4—H4A	109.3	C12—C13—C14	118.5 (3)
N5—C4—H4B	109.3	C12—C13—H13	120.8
C3—C4—H4B	109.3	C14—C13—H13	120.8
H4A—C4—H4B	108.0	N9—C14—C13	123.7 (3)
C8—N5—C4	111.4 (2)	N9—C14—H14	118.2
C8—N5—C6	110.4 (2)	C13—C14—H14	118.2
			
N9^i^—Cd1—S1—C1	178.45 (10)	C8—N5—C6—C7	−179.3 (2)
N9—Cd1—S1—C1	−98.07 (10)	C4—N5—C6—C7	−56.2 (3)
S1^i^—Cd1—S1—C1	40.12 (8)	C1—N2—C7—C6	105.6 (3)
S2—Cd1—S1—C1	−6.55 (8)	C3—N2—C7—C6	−59.0 (3)
S2^i^—Cd1—S1—C1	84.63 (9)	N5—C6—C7—N2	57.5 (3)
N9^i^—Cd1—S2—C1	20.45 (18)	N9^i^—Cd1—N9—C14	−49.4 (2)
N9—Cd1—S2—C1	100.31 (10)	S1^i^—Cd1—N9—C14	45.1 (2)
S1^i^—Cd1—S2—C1	−166.98 (8)	S1—Cd1—N9—C14	−140.6 (2)
S1—Cd1—S2—C1	6.58 (8)	S2—Cd1—N9—C14	151.7 (2)
S2^i^—Cd1—S2—C1	−98.30 (9)	S2^i^—Cd1—N9—C14	32.3 (3)
Cd1—S2—C1—N2	170.5 (2)	N9^i^—Cd1—N9—C10	133.4 (2)
Cd1—S2—C1—S1	−10.81 (13)	S1^i^—Cd1—N9—C10	−132.1 (2)
Cd1—S1—C1—N2	−170.4 (2)	S1—Cd1—N9—C10	42.2 (2)
Cd1—S1—C1—S2	10.87 (14)	S2—Cd1—N9—C10	−25.5 (2)
S2—C1—N2—C3	−9.9 (3)	S2^i^—Cd1—N9—C10	−144.91 (17)
S1—C1—N2—C3	171.4 (2)	C14—N9—C10—C11	−1.4 (4)
S2—C1—N2—C7	−172.5 (2)	Cd1—N9—C10—C11	175.9 (2)
S1—C1—N2—C7	8.8 (3)	N9—C10—C11—C12	1.2 (5)
C1—N2—C3—C4	−105.1 (3)	C10—C11—C12—C13	0.2 (5)
C7—N2—C3—C4	59.5 (3)	C11—C12—C13—C14	−1.3 (5)
N2—C3—C4—N5	−58.5 (3)	C10—N9—C14—C13	0.3 (4)
C3—C4—N5—C8	179.0 (2)	Cd1—N9—C14—C13	−177.1 (2)
C3—C4—N5—C6	56.5 (3)	C12—C13—C14—N9	1.1 (5)

Symmetry codes: (i) −*x*, *y*, −*z*+1/2.
